# Clinicopathologic and prognostic characteristics of alpha-fetoprotein–producing gastric cancer

**DOI:** 10.18632/oncotarget.15909

**Published:** 2017-03-06

**Authors:** Ruji He, Qinyi Yang, Xuqiang Dong, Yao Wang, Weiming Zhang, Lizong Shen, Zhihong Zhang

**Affiliations:** ^1^ Division of Gastrointestinal Surgery, Department of General Surgery, First Affiliated Hospital, Nanjing Medical University, Nanjing 210029, China; ^2^ Department of Pathology, First Affiliated Hospital, Nanjing Medical University, Nanjing 210029, China

**Keywords:** alpha-fetoprotein–producing gastric cancer, prognosis, risk factor, overall survival

## Abstract

Alpha-fetoprotein–producing gastric cancer (AFPGC) accounts for 1.5%–7.1% of all gastric cancer cases. Compared with other types of gastric cancer, AFPGC is more aggressive and prone to liver and lymph node (LN) metastasis, with extremely poor prognosis. To improve understanding of AFPGC we reviewed a consecutive series of 82 AFPGC patients and investigated the prognostic factors. The incidence of AFPGC among our gastric cancer patients was 1.95%, and 29.27% of AFPGCs were diagnosed with metastasis at the time of presentation, mainly liver metastasis. The serum AFP level of patients with AFPGC was significantly associated with tumor differentiation. Histologically, these AFPGC patients were composed of 34.55% hapatiod type, 58.18% fetal gastrointestinal type, 9.09% yolk sac tumor-like type, and 14.55% mixed type. Patient gender, tumor differentiation, Lauren classification, and number of metastatic lymph nodes showed significant differences among these four subtypes. The overall survival time was 42.02 months and the 3-year cumulative survival rate was 53.13%. Age, American Joint Committee on Cancer (AJCC) TNM staging classification (TNM stage), serum AFP level, and surgery were prognostic factors for overall survival; however, TNM stage was the only independent risk factor for prognosis of AFPGC. In short, AFPGC is a rare, unique, and heterogeneous entity, and its proper identification and treatment remain a challenge. More attention should be paid to AFPGC to improve patient care and the dismal prognosis.

## INTRODUCTION

Alpha-fetoprotein (AFP) is a well-known embryonal serum protein that is mainly produced by fetal liver cells and yolk sac cells [1] and commonly serves as an important tumor marker for hepatocellular carcinoma or yolk sac tumors. However, many studies have revealed that several other kinds of tumor can produce AFP, among which gastric cancer is the most common [2]. Gastric cancer with a high level of AFP is termed α-fetoprotein–producing gastric cancer (AFPGC) [3]. AFPGC was first described by Bourreille et al. in 1970 [4], and has since been reported all over the world but mostly in Asia, with an incidence of 1.5%–7.1% among all gastric cancer cases [2, 3, 5–8].

AFPGC shows aggressive characteristics and is prone to liver and lymph node metastasis; accordingly, AFPGC is known to have an extremely poor prognosis [8–11]. However, the pathogenesis and the standardized treatment process of AFPGC remain elusive [3], and most previous studies are case reports. To improve understanding of AFPGC we retrospectively reviewed the clinicopathologic features of a consecutive series of 82 AFPGC patients in the First Affiliated Hospital, Nanjing Medical University, and investigated prognostic factors. We found that the serum AFP level of patients with AFPGC was significantly associated with tumor differentiation, and that patient gender, tumor differentiation, Lauren classification, and number of metastatic lymph nodes showed significant association with the four subtypes of AFPGC. The overall survival time of the 72 AFPGC patients with follow-up was 42.02 months, and the 3-year cumulative survival rate was 53.13%. Age, TNM stage, serum AFP level, and surgery were prognostic factors for overall survival; however, TNM stage was the only independent risk factor for prognosis of AFPGC.

## RESULTS

### General characteristics

The 82 study patients with AFPGC have elevated serum AFP level, ranging from 20.5 ng/ml to more than 1,210 ng/ml with a median of 73.2 ng/ml. These patients included 62 males and 20 females with a median age of 62.5 years (range, 22–78 years). Regarding tumor location, 31 had tumor in the upper third of the stomach, 11 in the middle third, and 22 in the distal third, 17 patients had more than two thirds of the stomach affected, and one patient had gastric cancer with undetermined location.

Sixty-nine (84.15%) of our study patients underwent surgical treatment: of these, 54 patients underwent radical D2 gastrectomy including 36 cases of radical total gastrectomy, 16 cases of radical distal gastrectomy, and two cases of radical proximal gastrectomy. Six patients underwent curative-intent gastrectomy combined with other organ resection, including two cases of total gastrectomy with partial hepatectomy (for liver metastasis), two cases of distal gastrectomy with partial hepatectomy (for liver metastasis), one case of total gastrectomy with right ovariectomy (for ovarian metastasis), and one case of total gastrectomy with splenectomy (for N11 lymph node metastasis). Six patients with M1 disease underwent palliative gastrectomy, including two cases of total gastrectomy, one case of proximal gastrectomy, one case of distal gastrectomy, and two cases of gastrojejunostomy. Three cases were confirmed as T4b disease to pancreas and were treated with palliative total gastrectomy, palliative distal gastrectomy, and only laparotomy respectively.

Of the 82 patients with AFPGC, 24 cases (29.27%) were diagnosed with metastasis (M1) at the time of presentation including 17 cases of liver metastasis, two cases of ovarian metastasis, three cases of omentum metastasis, and two cases of other organ metastasis. Of these 24 cases, 12 did not undergo surgical intervention.

Thirty-four patients (47.22%) who underwent surgical treatment were followed up for serum AFP level. The serum AFP level decreased after radical surgical treatment in 28 patients and usually returned to a normal level in postoperative 1–3 months. Among six cases with persistently elevated postoperative serum AFP level, four patients underwent only palliative surgery and the underlying reason was not determined in the other two cases.

We analyzed the correlation between preoperative serum AFP level and clinicopathologic features in these AFPGC patients. As shown in Table 1, the preoperative serum AFP level showed no significant association with gender, age, tumor location, TNM stage, or liver metastasis. However, the serum AFP level in patients with poorly differentiated tumor was significantly higher than that in patients with well-differentiated tumor (median 97.03 ng/ml *vs*. 36.38 ng/ml, *P*=0.0268). We also evaluated the relationship between the preoperative serum AFP and carcinoembryonic antigen (CEA) level, and found no significant association (*P*=0.1979).

**Table 1:** Correlation of serum alpha-fetoprotein (AFP) level with clinicopathologic features in AFPGC patients

Clinicopathological features	N	Serum AFP (ng/ml)[median (range)]	*P* value
Gender			0.070
Male	62	93.22(44.5625-823.825)	
Female	20	54.75(31.275-172.825)	
Age			0.909
<60yo	26	72.00(39.375-339.45)	
≥60yo	56	73.20(39.65-439.875)	
Location			0.483
Upper third	31	54.10 (36.38-450.40)	
Middle third	11	45.55 (28.25-310.80)	
Lower third	23	94.16 (57.07-847.00)	
Two thirds or more	17	60.80 (50.40-476.10)	
Differentiation			0.031
Well differentiated	7	36.38 (24.00-46.30)	
Poorly differentiated	64	97.03 (46.425-622.375)	
T stage			0.618
T1	5	39.20 (24.30-271.35)	
T2	6	77.70 (41.9375-335.175)	
T3	3	74	
T4	54	83.34 (37.22-850.45)	
N stage			0.770
N0	8	45.925 (34.05-354.075)	
N1	11	74.00 (33.30-393.90)	
N2	18	122.25 (49.95-850.45)	
N3	30	83.34 (32.795-759.225)	
M stage			0.811
M0	58	73.20 (35.825-418.825)	
M1	24	76.30 (46.425-223.15)	
TNM stage			0.301
I~II	14	45.925 (30.075-208.35)	
III	42	96.09 (35.825-823.825)	
IV	24	76.30 (46.425-223.15)	
Liver metastasis			0.567
Yes	17	83.00 (47.15-540.50)	
No	65	72.40 (36.94-401.10)	

### Clinicopathologic characteristics of four morphologic subtypes of AFPGC

**Figure 1:**
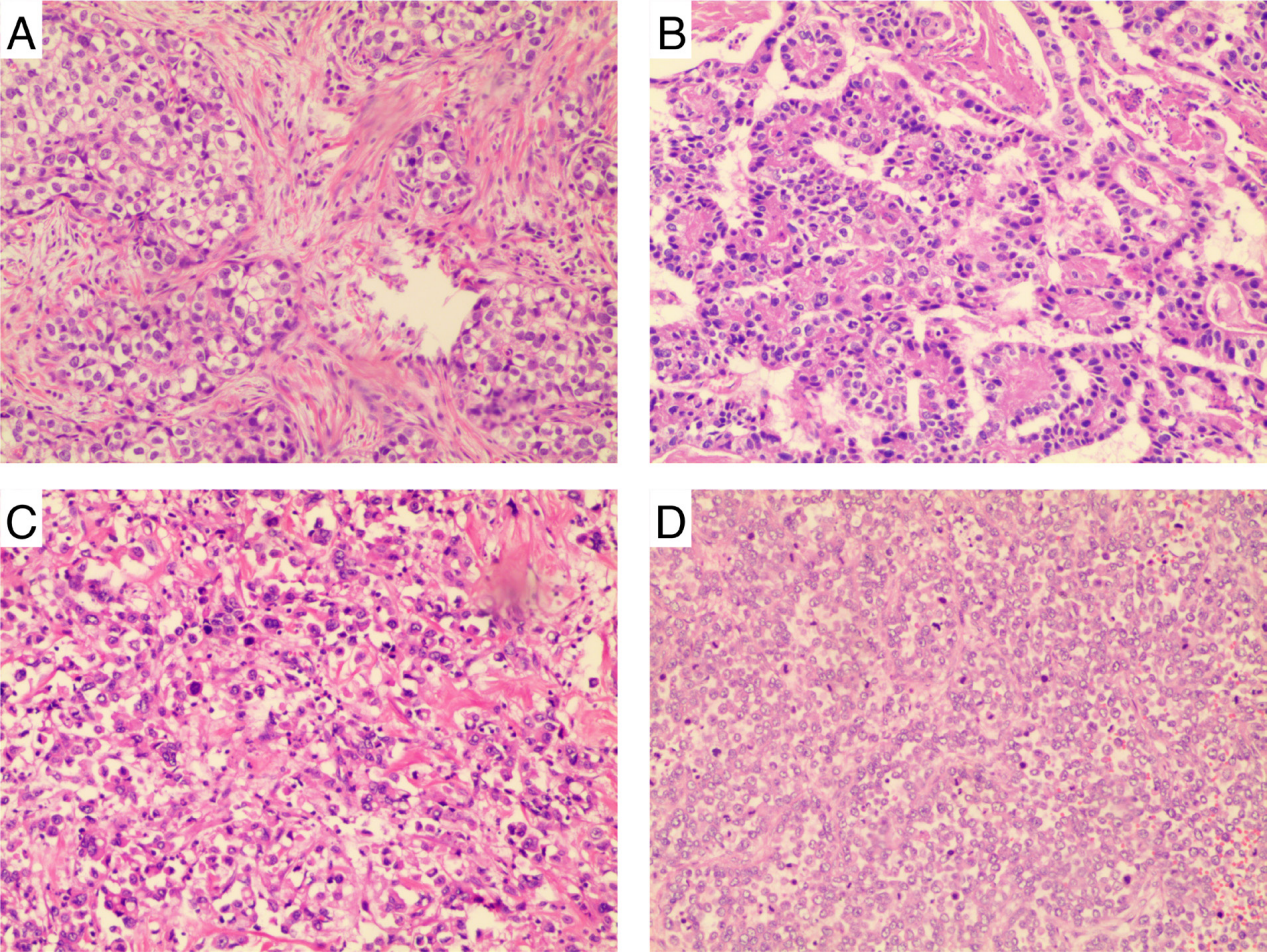
Histologic subtypes of AFPGC **(A)** hepatoid type. Large polygonal hepatocyte-like cells with clear cytoplasm, resembling metastatic hepatocellular carcinoma but without biles. **(B)** fetal gastrointestinal type. Neoplastic glandular like early gut origin adenocarcinoma. **(C)** yolk sac tumor-like type. Reticular patterns formed by a loose network of sheets or nests with flat or cuboidal cells; **(D)** mixed type. Pleomorphic cells formed glandular clefts. Original magnification, ×100.

According to previous studies [10, 12–15], the morphologic spectrum of AFPGC includes four subtypes: hepatoid type, fetal gastrointestinal type, yolk sac tumor-like type, and mixed type (Figure 1). On pathologic review, the 55 AFPGC patients with complete pathologic data consisted of 19 cases of hapatoid type (34.55%), 32 fetal gastrointestinal type (58.18%), 5 yolk sac tumor-like type (9.09%), and 8 mixed type (14.55%). We analyzed the correlation between subtype and clinicopathologic features, and further investigated the expression of AFP, vascular endothelial growth factor (VEGF), and CEA in these tumor tissues using immunohistochemistry. As shown in Table 2, patient gender, tumor differentiation, Lauren classification, and number of lymph node (LN) metastases showed significant association with the four subtypes. However, TNM stage, liver metastasis, serum AFP level, and tumor AFP or CEA immunoreactivity did not present a correlation with these subtypes.

**Table 2:** Clinicopathologic characteristics of four subtypes of AFPGC

Variables	Hepatoid type(n=19)	Fetal gastrointestinal type(n=32)	Yolk sac tumor-like type(n=5)	Mixed type(n=8)	Chi-square	*P* value
Gender					10.143	0.010
Male	16	26	4	2		
Female	3	6	1	6		
Age					2.118	0.595
<60yo	8	8	1	3		
≥60yo	11	24	4	5		
Tumor location					7.925	0.522
Upper third	7	13	2	2		
Middle third	4	4	0	2		
Lower third	5	9	1	0		
Two-thirds or more	3	6	2	4		
Histological differentiation					7.860	0.047
Well differentiated	0	7	0	0		
Poorly differentiated	19	25	5	8		
Lauren classification					46.802	0.000
Intestinal	19	32	0	2		
Diffuse	0	0	5	2		
Mixed	0	0	0	4		
Depth of invasion					9.489	0.264
T1	2	3	0	0		
T2	3	2	0	1		
T3	1	0	0	2		
T4	13	27	5	5		
N status					8.282	0.462
N0	3	3	0	2		
N1	3	7	0	1		
N2	5	11	0	1		
N3	8	11	5	4		
TNM stage					4.127	0.947
I	3	3	0	1		
II	2	3	0	2		
III	12	20	4	4		
IV	2	6	1	1		
Lymphovascular invasion					3.649	0.324
Positive	10	9	2	4		
Negative	9	23	3	4		
Nerve invasion					3.854	0.272
Positive	3	8	3	2		
Negative	16	24	2	6		
Liver metastasis					1.318	0.803
Yes	2	5	0	0		
No	17	27	5	8		
Serum AFP level					4.990	0.167
<200ng/ml	8	23	3	6		
≥200ng/ml	11	9	2	2		
AFP immunoreactivity in tumor					3.234	0.354
-	12	25	5	7		
+~++	7	7	0	1		
VEGF immunoreactivity in tumor					2.543	0.466
-~+	10	23	4	6		
++	9	9	1	2		
CEA immunoreactivity in tumor					10.945	0.065
-	12	8	1	1		
+	4	13	1	4		
++~+++	3	11	3	3		

Variables	Hepatoid type(n=19)	Fetal gastrointestinal type(n=32)	Yolk sac tumor-like type(n=5)	Mixed type(n=8)	*F* value	*P* value
Average tumor size (cm)	5.58±2.957	5.55±2.377	8.80±4.087	7.36±5.344	2.124	0.107
LN harvest	27.11±14.541	24.69±11.893	31.00±8.803	32.50±14.283	0.992	0.403
No. of LN metastasis	8.95±9.600	7.75±8.394	21.60±7.403	9.25±8.598	3.648	0.017
Serum AFP (ng/ml)	525.30±494.093	275.05±411.640	513.86±595.921	153.26±227.547	2.112	0.108
Serum CEA (ng/ml)	23.72±48.241	75.22±165.076	3.65±1.279	11.47±14.125	0.856	0.473

### Survival analysis

**Figure 2:**
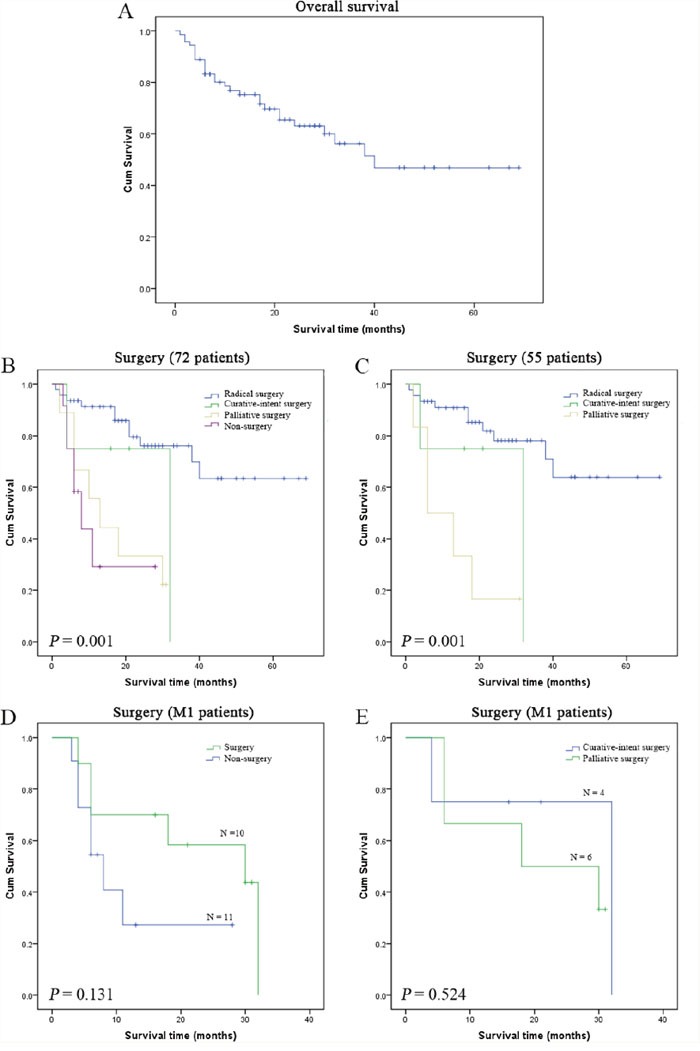
Survival analysis for AFPGC patients The mean overall survival (OS) of the 72 patients was 42.02 months, and the 3-year cumulative survival rate was 53.13% **(A)**, and surgical treatment showed significant beneficial effects on OS of 72 AFPGC patients (**(B)**, *P*=0.001). Compared with palliative surgery, radical surgery and curative-intent surgery produced significant survival benefits for 55 AFPGC patients with complete clinicopathologic data (**(C)**, *P*=0.001). However, surgical treatment did not produce survival benefit for 21 AFPGC patients with synchronous M1 disease (**(D)**, *P*=0.131), and curative-intent surgery also did not have survival advantage over palliative surgery in these M1 patients (**(E)**, *P*=0.524).

Although all patients received follow-up, 10 patients (12.2%) were lost to follow-up. As shown in Figure 2A, the overall survival time (OS) of the remaining 72 patients was 1–69 months (mean 42.02 months) and the 3-year cumulative survival rate of the 72 patients was 53.13%.

The survival time of the surgery group (including radical gastrectomy, curative-intent gastrectomy combined with other organ resection, palliative gastrectomy, gastrojejunostomy, and laparotomy) was 1–69 months (mean 45.43 months) and the 3-year survival rate was 58.10%; in contrast, the survival time for the non-surgery group was only 4–28 months (mean 12.85 months) (Figure 2B, *P*=0.001). Furthermore, radical surgery and curative-intent surgery could produce significant survival benefits for 55 AFPGC patients with complete clinicopatholigic data with comparison to palliative surgery (Figure 2C, *P*=0.001). For 21 patients with synchronous M1 disease who had complete follow-up data there was no survival benefit from surgical treatment; the survival time was 4–32 months (mean 22.08 months) in the surgery group and 3–28 months (mean 12.32 months) in the non-surgery group (Figure 2D, *P*=0.131). Moreover curative-intent surgery for M1 disease did not show a survival advantage over palliative surgery (mean 25.00 months *vs*. 20.33 months, *P*=0.524) (Figure 2E).

The Kaplan–Meier method and log-rank test for 72 patients showed significant survival differences according to age, TNM stage (especially M0 *vs*. M1 and presence *vs*. absence of liver metastasis), and surgery (Table 3). Similar results were observed for the 55 AFPGC patients with complete pathologic data and tests in this subgroup indicated that serum AFP level (<200 ng/ml *vs*. ≥200 ng/ml) was a prognostic factor for overall survival (*P*=0.030) (Table 4). However, multivariate Cox regression analysis showed that only TNM stage was an independent risk factor for prognosis in AFPGC (Table 5).

**Table 3:** Prognostic factors for overall survival analyzed by the Kaplan–Meier method in 72 AFPGC patients with follow-up

Variables	Total No.	No. of events	Means for survival time (months)				Chi-square	*P* value
			Estimate	Std. Error	%95 CI			
					Lower	Upper		
Gender							2.073	0.150
Male	56	19	43.814	4.377	35.235	52.394		
Female	16	8	34.222	7.162	20.184	48.260		
Age							4.047	0.044
<60yo	20	11	27.908	6.268	15.621	40.194		
≥60yo	52	16	46.178	4.229	37.889	54.467		
Location							2.625	0.453
Upper third	27	9	37.137	6.631	24.140	50.133		
Middle third	9	2	49.733	8.064	33.929	65.538		
Lower third	22	10	39.871	6.468	27.193	52.549		
Two thirds or more	14	6	27.136	5.564	16.230	38.041		
Differentiation							0.035	0.851
Well differentiated	7	2	38.286	6.754	25.047	51.524		
Poorly differentiated	55	19	44.712	4.300	36.283	53.140		
Lauren classification							1.469	0.480
Diffuse type	4	2	20.750	11.438	0.000	43.169		
Intestinal type	47	14	47.726	4.567	38.774	56.678		
Mixed type	4	1	17.500	0.354	16.807	18.193		
M stage							12.245	0.000
M0	51	14	49.321	4.304	40.884	57.757		
M1	21	13	17.904	2.936	12.150	23.658		
TNM stage							14.756	0.001
I~II	14	1	64.143	4.680	54.969	73.317		
III	35	12	44.620	4.956	34.906	54.335		
IV	21	13	17.904	2.936	12.150	23.658		
Liver metastasis							6.420	0.011
Yes	16	9	18.503	3.496	1.651	25.354		
No	56	18	46.288	4.225	38.007	54.570		
Vascular or lymphatic invasion							0.020	0.887
Yes	22	7	48.038	6.492	35.313	60.763		
No	35	11	42.711	4.585	33.723	51.698		
Nerve invasion							0.314	0.575
Yes	14	5	42.151	7.373	27.699	56.603		
No	43	13	47.450	4.706	38.227	56.674		
Serological AFP level							1.683	0.195
<200ng/ml	47	14	47.886	4.695	38.683	57.088		
≥200ng/ml	25	13	34.348	5.548	23.474	45.223		
Surgery							22.052	0.000
Radical surgery	47	11	52.110	4.283	43.716	60.504		
Curative-intent surgery	4	2	25.000	8.573	8.197	41.803		
Palliative surgery	9	7	16.333	3.668	9.143	23.523		
No surgery	12	7	12.854	3.388	6.213	19.495		

**Table 4:** Prognostic factors for overall survival analyzed by the Kaplan–Meier method in 55 AFPGC patients with complete clinicopathologic data

Variables	Total No.	No. of events	Means for survival time (months)				Chi-square	*P* value
			Estimate	Std. Error	%95 CI			
					Lower	Upper		
Gender							0.187	0.666
Male	43	13	46.749	4.825	37.291	56.206		
Female	12	4	43.562	7.922	28.035	59.090		
Age							5.431	0.020
<60yo	14	8	29.785	6.943	16.176	43.393		
≥60yo	41	9	49.255	3.990	41.434	57.075		
Location							4.070	0.254
Upper third	21	7	31.310	3.321	24.801	37.818		
Middle third	8	1	55.200	6.977	41.526	68.874		
Lower third	14	4	51.016	7.394	36.524	65.508		
Two thirds or more	12	5	28.333	5.841	16.884	39.783		
Differentiation							0.000	0.995
Well differentiated	7	2	38.286	6.754	25.047	51.524		
Poorly differentiated	48	15	47.041	4.561	38.100	55.981		
Lauren classification							1.469	0.480
Diffuse type	4	2	20.750	11.438	0.000	43.169		
Intestinal type	47	14	47.726	4.567	38.774	56.678		
Mixed type	4	1	17.500	0.354	16.807	18.193		
Morphologic subtype							2.589	0.459
Hapatiod	15	3	55.780	6.794	42.463	69.097		
fetal gastrointestinal	30	11	36.780	3.988	28.963	44.596		
yolk sac tumor-like	4	2	20.750	51.438	0.000	43.169		
mixed	6	1	51.500	9.959	31.980	71.020		
M stage							6.089	0.014
M0	47	12	50.468	4.461	41.725	59.211		
M1	8	5	19.813	4.919	10.172	29.453		
TNM stage							8.329	0.016
I~II	14	1	64.143	4.680	54.969	73.317		
III	33	11	42.532	4.747	33.229	51.835		
IV	8	5	19.813	4.919	10.172	29.453		
Liver metastasis							3.388	0.066
Yes	7	4	21.786	5.270	11.457	32.115		
No	48	13	49.518	4.466	40.764	58.272		
Vascular invasion							0.012	0.911
Yes	20	6	49.261	6.723	36.084	62.438		
No	35	11	42.711	4.585	33.723	51.698		
Nerve invasion							0.036	0.849
Yes	13	4	44.962	7.482	30.297	59.626		
No	42	13	46.495	4.855	36.980	56.011		
AFP immunoreactivity in tumor							0.619	0.431
-	42	14	44.952	5.029	35.095	54.809		
+, ++	13	3	41.046	5.182	30.890	51.202		
CEA immunoreactivity in tumor							0.144	0.931
-	20	6	47.874	6.993	34.168	61.579		
+	20	6	40.626	4.714	31.386	49.866		
++, +++	15	5	43.018	7.079	29.144	56.893		
VEGF immunoreactivity in tumor							0.084	0.773
-, +	38	11	48.209	5.158	38.100	58.318		
++	17	6	36.536	4.715	27.295	45.776		
Serum AFP level							4.724	0.030
<200ng/ml	34	6	56.884	4.492	48.079	65.688		
≥200ng/ml	21	11	30.739	4.031	22.838	38.640		
Surgery							16.847	0.000
Radical surgery	45	10	52.537	4.426	43.861	61.212		
Curative-intent surgery	4	2	25.000	8.573	8.197	41.803		
Palliative surgery	6	5	12.667	3.970	4.886	20.447		

**Table 5:** Multivariate analysis by the Cox model

Clinicopathological factors	Covariate Means	*P* value	RR	95% CI for *RR*	
				Lower	Upper
Age	0.743	0.133	0.534	0.236	1.210
TNM stage		0.002			
III vs I~II	0.500	0.298	3.023	0.376	24.301
IV vs I~II	0.300	0.021	11.441	1.433	91.359
Serum AFP	0.343	0.065	2.179	0.953	4.983
Surgery	0.157	0.080	3.088	0.873	10.926

*RR*: relative risk.

## DISCUSSION

Alpha-fetoprotein–producing gastric cancer is a relatively rare form of gastric cancer. In the present study AFPGC accounted for approximately 1.95% of all gastric cancers, which is similar to previous reports [2, 3, 5–8]. Van der Veek et al. [17] proposed that AFP values greater than 500 ng/ml are unlikely to be due to benign conditions, but only 18.29% (15/82) of AFPGC patients in the present series met this criterion. More importantly, among the AFPGC patients who were followed for serum AFP level, serum AFP level decreased rapidly after radical operation but remained high level after palliative surgery, strongly suggesting that AFP was produced by gastric cancer cells [2]. In this study, serum AFP level was significantly associated with tumor differentiation; however, no correlation with metastasis or liver metastasis was found [11].

Liver metastasis is a characteristic feature of AFPGC, occurring in 33%–72% of all AFPGC cases [9, 17]. Twenty-four patients (29.27%) in our study were diagnosed as synchronous metastasis, and most of these were liver metastasis, but we did not obtain details of metachronous liver metastasis or other metastasis during follow-up. Metachronous liver metastasis may occur in approximately 50% of patients who undergo curative resection of the tumor within a year after surgery [17, 18], and elevation of serum AFP level may be detected prior to appearance of symptoms and imaging detection. Therefore, AFPGC patients should be carefully observed for early detection and treatment of possible recurrent disease by measuring the serum AFP levels as a follow-up marker [9].

AFPGC can be divided into four morphologic subtypes. The hepatoid type and the yolk sac tumor-like type are derived from liver cell metaplasia and yolk sac cell metaplasia of common poorly differentiated medullary adenocarcinoma, respectively, whereas the fetal gastrointestinal type appears to be imitation of fetal gastrointestinal epithelium by common tubular adenocarcinoma [12]. Unlike previous reports [10, 12], the hepatoid type only accounted for 34.55% of the AFPGC cases in this study whereas more than half of our cases were the fetal gastrointestinal type. Tsung proposed that the criterion for diagnosing AFPGC is positive staining of AFP in the primary lesion by immunohistochemical methods [6]. However, the incidence of AFP-positive expression in this study was only 27.27% (Table 2, 15/55), which was lower than that in other studies [10, 15, 19]. The expression of VEGF and CEA was also evaluated by immunohistochemistry, and our results indicated that AFP immunoreactivity was more common in the hepatoid type (Table 2, 7/12, 36.84%) whereas CEA was more common in the fetal gastrointestinal type (Table 2, 24/32, 75%), which is consistent with findings of previous studies [10, 17]. There were no significant differences in AFP, VEGF, and CEA expression among these four morphologic subtypes. All hepatoid type AFPGCs were poorly differentiated and had a higher incidence of lymphovascular invasion indicating that the hepatoid type is highly malignant [12], although there was no survival difference among the four subtypes. This study also showed that the yolk sac tumor-like type of AFPGC might be the most detrimental type; all tumors of this type were poorly differentiated and diffuse type of Lauren classification and had the highest number of metastatic lymph nodes. Wang et al. [10] also revealed that the yolk sac tumor-like type AFPGC had the worst prognosis among the four subtypes although no significant survival difference was found.

AFPGC has been considered to have unfavorable long-term survival, mainly due to the higher incidence of liver metastasis and lymphovascular invasion [9, 20]. In this study, the mean survival time was 42.02 months and the 3-year cumulative survival rate was 53.13%. Shibata et al. reported that the 5-year survival rate post-curative resection is only 8.3% [9]. The mechanisms underlying the poor prognosis are not well understood although AFP has been reported to have a suppressive effect on lymphocyte transformation [20], to enhance tumor cell proliferation through the HGF and c-Met pathway [9, 21], and to increase angiogenesis via VEGF expression [22, 23].

In the present report, patient age, TNM stage, and surgical treatment were found to be associated with overall survival. It is easily understood that young AFPGC patients are prone to a more detrimental prognosis. Metastatic diseases, especially liver metastasis, and advanced TNM stage were significantly negatively correlated with survival time, consistent with many previous reports [10, 11]. It is interesting that serum AFP level was a risk factor for survival in a subgroup of these patients, and the mean survival time of patients with AFP level greater than 200 ng/ml was shorter than that in patients with AFP level lower than 200 ng/ml (Table 4). To our best knowledge, this is the first report of a significant association between serum AFP levels and overall survival in AFPGC patients. More than 80% of these patients underwent surgical treatment. Surgical treatment, especially radical surgery and curative-intent surgery, could produce survival advantages. However, surgical treatment for M1 disease did not present a survival benefit and curative-intent surgery was also not favorable for these patients, indicating that it will be important to develop a novel effective multimodal therapy for AFPGC [6, 9]. However, multivariate analysis indicated that only TNM stage was an independent risk factor for prognosis in AFPGC, consistent with previous studies [10].

Obviously, limited knowledge and limited successful treatment options exist for AFPGC [17]. It has gradually become recognized that AFPGC is quite different from the conventional type of gastric cancer. Recently, several studies have been conducted on AFPGC with the aim of improving the outcome of AFPGC patients. He et al. [8] investigated the differential expression of proteins between AFPGC and AFP non-producing gastric cancer and found that high level expressions of XIAP and IGF-Irβ in tumor tissues were independent factors for poor prognosis in AFPGC patients, and that AFPGC may be separated into two subgroups with involvement of a distinct set of signaling pathways based on a risk model of XIAP and IGF-Irβ expression and TNM stage. Shimakata et al. [7] investigated expression levels of a panel of solute carrier transporters (SLC) in AFPGC and conventional gastric cancer and proposed that patients with AFPGC may potentially benefit from gemcitabine/fluoropyrimidine combination chemotherapy. Better understanding of AFPGC at the cellular and molecular levels will aid the development of individualized therapy for AFPGC [3, 6].

In conclusion, AFPGC is a rare, unique, and heterogeneous entity, and its proper identification and treatment remain a challenge. The present study helps us understand AFPGC; however, greater attention must be paid to AFPGC to improve patient care and the dismal prognosis.

## MATERIALS AND METHODS

### Patients

Approximately 4,200 patients with primary gastric adenocarcinoma were diagnosed and treated in the First Affiliated Hospital, Nanjing Medical University from January 2010 to May 2016. We searched for patients with elevated serum alpha-fetoprotein (AFP) level on admission among these cases. Serum AFP level was determined by ELISA, and a concentration greater than 20 ng/ml was considered elevated. Patients with abnormal liver function or with liver diseases such as acute or chronic hepatitis, cirrhosis, fatty liver, alcoholic liver, and primary liver cancer were excluded. A total of 82 patients (1.95%) were confirmed as alpha-fetoprotein–producing gastric cancer (AFPGC) and were enrolled in the present study. All patients were diagnosed pathologically according to the AJCC criteria (7th ed., 2010). The clinicopathologic data of these enrolled patients were collected retrospectively and the patients received follow-up for survival by telephone or subsequent consultation with a cut-off date of October 2016. The follow up time was 4–70 months (median: 27.5 months). Patients provided their written informed consent. Samples were stored in the hospital database for studies. This study was approved by the Nanjing Medical University Institutional Review Board, and complied with the Helsinki Declaration.

### Tissue array and immunohistochemistry

All pathologic data of the enrolled patients were reviewed independently by two experienced pathologists for histologic classification according to previous reports [10, 13].

Paraffin-embedded formalin-fixed tissues were obtained and re-embedded in an arrayed master block. The Beecher Instruments arraying device (Sun Prairie, WI, USA) was used to produce circular sample spots 1.0 mm in diameter. Immunohistochemistry was performed according to standard protocols. Briefly, 4-μm sections were deparaffinized in xylene and rehydrated in graded ethanol. Antigen retrieval was achieved by boiling the sections in 10 mM citrate buffer (pH 6.0) for 10 min in a pressure cooker. The sections were then sequentially blocked with 3% hydrogen peroxide/methanol for 10 min and with non-immune serum for 10 min. The sections were incubated overnight at 4°C with primary antibodies against AFP, CEA, and VEGF (Fuzhou Maixin Biotechnology, Fuzhou, China). Negative controls were prepared by omitting the primary antibody. After a wash with phosphate-buffered saline (PBS), the sections were incubated with biotin secondary antigen for 15 min at 37°C and then the chromogen was developed for 5 min with liquid 3,3′-diaminobenzidine. Nuclei were counterstained with hematoxylin.

The staining was evaluated by two pathologists on the basis of the percentage of stained cells and staining density. The grades for the percentage of stained cells ranged from 0 to 4 (0, unstained cells; 1, 1–10% stained cells; 2, 11–50% stained cells; 3, 51–80% stained cells; 4, 81–100% stained cells). The grades for staining density ranged from 0 to 3 (0, unstained cells; 1, slightly stained cells; 2, moderately stained cells; 3, highly stained cells). The two scores were multiplied, resulting in the following levels of immunohistochemical staining: 0 points, −; 1–4 points, +; 5–8 points, ++; and 9–12 points, +++.

### Statistical analysis

Statistical analysis was performed using SPSS 22.0 software (SPSS Inc., IL, USA). Mann–Whitney U test and Kruskal–Wallis test were used to analyze the relationship between serum AFP level and clinicopathologic features. The Chi-square Test, Fisher Probabilistic Methods, and ANOVA were used to analyze the relationships between the four subtypes of AFPGC and clinicopathologic features. The clinicopathologic factors were analyzed by the Kaplan–Meier method and compared by the log-rank test. The Cox model was also used to analyze the prognostic factors in a multivariate analysis. The observed end point was death. All tests were two-sided. A *P* value <0.05 was considered statistically significant.

## Abbreviations

AFPGC: α-fetoprotein–producing gastric cancer
AFP: α-fetoprotein
VEGF: vascular endothelial growth factor
CEA: carcinoembryonic antigen
OS: overall survival
LN: lymph node
